# Coronaviruses in Brazilian bats: A matter of concern?

**DOI:** 10.1371/journal.pntd.0008820

**Published:** 2020-10-26

**Authors:** Samuel Cibulski, Francisco Esmaile Sales de Lima, Paulo Michel Roehe

**Affiliations:** 1 Centro de Biotecnologia–CBiotec, Laboratório de Biotecnologia Celular e Molecular, Universidade Federal da Paraíba–UFPB, João Pessoa, Paraíba, Brazil; 2 Faculdade de Medicina Veterinária, Universidade de Fortaleza–UNIFOR, Fortaleza, Ceará, Brazil; 3 Departamento de Microbiologia Imunologia e Parasitologia, Laboratório de Virologia, Universidade Federal do Rio Grande do Sul–UFRGS, Porto Alegre, Porto Alegre, Rio Grande do Sul, Brazil; Instituto de Pesquisas Veterinarias Desiderio Finamor, BRAZIL

According to the United Nations Environment Programme (UNEP), Brazil is the most biologically diverse country in the world. It is estimated that Brazil hosts between 15% and 20% of the Earth’s biological diversity, with the greatest number of endemic species on a global scale. Taking into account its wide natural diversity, Brazil suffers from serious environmental troubles. These problems, such as deforestation—leading to fragmentation and loss of habitats and overexploitation of plants, animals, and monoculture by agribusiness—are the main threats to Brazilian biodiversity [1]. Despite the plenteousness of legislation aiming protection and preservation of the natural environment, the continuing destruction of biomes is still an ongoing matter of concern [2].

The invasion of natural ecosystems facilitates the contact between people, domestic animals, and wildlife populations. Indigenous, isolated populations are at increasing risk of acquiring infectious agents to which they have not been previously exposed. The currently ongoing pandemic caused by Severe Acute Respiratory Syndrome Coronavirus 2 (SARS-CoV-2) plus the past outbreaks of Severe Acute Respiratory Syndrome (SARS) and Middle East Respiratory Syndrome (MERS) have once more highlighted (flipped the coin to reveal) how devastating and life threatening viral infections could be.

Multiple lines of evidence support an evolutionary origin for all human coronaviruses (CoV) from wild hosts, especially bats, which, due to its extraordinary adaptability to different niches, host a variety of microorganisms. Given the proper occasion, genetic diversity will play its part in evolution and eventually give rise to infectious agents, which could jump to a different animal species with unpredictable consequences. Bats are known to be reservoirs of a number of viruses with the potential to cause serious human diseases, including rabies, Ebola, Nipah, and the currently pandemic Coronavirus Disease 2019 (COVID-19), caused by SARS-CoV-2.

There is a consensus that the question is not “if” novel zoonotic viruses will rise but “when.” Currently, 2 to 3 novel RNA viruses are discovered every year; RNA viruses are the most common class of pathogens behind new human diseases [3]. Central and South America are considered world hotspots for the emergence of new mammalian viral zoonoses [4]. Brazil is a country of continental dimensions and home to an immense biodiversity and with more than 200 million inhabitants; with ecosystems constantly threatened by degradation, plus human and wildlife in close contact in several locations, these facilitate jumps of infectious agents between species.

Brazil is the country with the greatest diversity of mammals, with approximately 700 species, of which about 25% are bats [5]. At least 178 species of bats have been identified in different biomes, some of which definitely associated with the transmission of important zoonoses, such as rabies and histoplasmosis. Despite the large number of bat species, studies that explore the microbiomes of such species are scarce. Nevertheless, a few have revealed potential risks (e.g., pathogen spillover and/or transmission), such as CoV and representatives of other viral families in both synanthropic and sylvatic bats [6–9]. Members of the family Coronaviridae infect mammals, birds, and fish and are allocated into 4 genera: *Alphacoronavirus*, *Betacoronavirus*, *Gammacoronavirus*, and *Deltacoronavirus* (colloquially, alpha-, beta-, gamma-, and deltacoronaviruses). Due to the great diversity of alphacoronaviruses and betacoronaviruses reported in bats, it is believed that such species are reservoirs and progenitors of many members of these genera, whereas delta- and gammacoronaviruses seem to have originated from CoV of birds. The emergence of SARS-CoV-2 has thrusted bat betacoronaviruses into the spotlight again. Most CoV recovered from Brazilian bats are alphacoronaviruses [6,7,10]. However, in 2016, 2 distinct betacoronaviruses that clustered within MERS-CoV were detected in bats with potential contact with humans from the Atlantic Forest biome [11]. Other betacoronaviruses were reported in Central and South America [8,9], highlighting the broad circulation of these viruses in neotropical bats. Furthermore, the diversified ecology, a high number of coexisting bat species and their local abundance in relation to other mammalian species could make neotropical bats major spreaders of viruses, as human population density increases in the ever growing occupation of neotropical ecosystems [9].

Should CoV in Brazilian bats be a matter of concern? A prudent answer to this question would certainly be positive. However, unfortunately, it is not possible to calculate the risk and/or the point in time where viral host switching events might occur; moreover, even more difficult would be to predict the emergence of an outbreak or epidemics. Nonetheless, it is possible to state categorically that this will happen, as we have seen with SARS-CoV-2 and many other emergent viruses that spilled over from wildlife with impact on human health in the last 20 years. Furthermore, the scarce but valuable data on viral detection in neotropical bats reinforce the need for expanded and continuing efforts to widen our knowledge on the viral communities of wild and synanthropic mammals. This will certainly contribute to contingency plans in the advent of potential viral threats to humans and/or other species.

How can we be prepared? Is there anything we can do to minimize the burden of such inevitable course? The answer, if it exists, is not simplistic. Improvements on the national health system (Sistema Único de Saúde (SUS))—to ensure sufficient and adequate access to healthcare, allied to a strong focus on environmental education since the very early days of school attendance—would certainly have a positive effect on the formation of individuals concerned with environmental problems. Moreover, investments on research on the microbiome of wild and synanthropic animals may increase our degree of preparedness and contribute to contingency plans to reduce the potential risk of zoonotic diseases. However, the preventive measure that might have the most significant impact the emergence of new, potentially zoonotic viruses is the implementation of conservation policies to control the disturbance of biomes. Ensuring environmental interventions along with sustainable exploration of natural resources would minimize exposures to risk factors from emerging wildlife diseases, yet preserving wildlife in its natural “status quo,” thus contributing indirectly to accomplish the interrelated goals of health, environmental sustainability, and development.

Such achievement would require more profound interventions, where the One Health concept spanning human, animal, and environmental health would become part of a unified effort, stimulating inter- and multidisciplinary professional, disciplinary, and institutional bonds to work in a more integrated fashion [12]. A complex concern such as the emergence of infectious diseases, especially in low-income countries, needs to be addressed using this key concept, in which particular characteristics of human, environmental, and animal health are considered in a unified way to more effectively detect, understand, prevent and, if necessary, to intervene aiming to solve related public health issues [2,13–16].

## References

[1] Haddad NM, Brudvig LA, Clobert J, Davies KF, Gonzalez A, Holt RD et al Habitat fragmentation and its lasting impact on Earth’s ecosystems. Sci Adv. 2015 10.1126/sciadv.1500052 26601154 PMC4643828

[2] Ellwanger JH, Kulmann-Leal B, Kaminski VL, Valverde-Villegas JM, DA VEIGA ABG, Spilki FR et al Beyond diversity loss and climate change: Impacts of Amazon deforestation on infectious diseases and public health. An Acad Bras Cienc. 2020 10.1590/0001-3765202020191375 32321030

[3] Rosenberg R. Detecting the emergence of novel, zoonotic viruses pathogenic to humans. Cell Mol Life Sci. 2015 10.1007/s00018-014-1785-y 25416679 PMC4629502

[4] Olival KJ, Hosseini PR, Zambrana-Torrelio C, Ross N, Bogich TL, Daszak P. Host and viral traits predict zoonotic spillover from mammals. Nature. 2017 10.1038/nature22975 28636590 PMC5570460

[5] Nogueira MR, de Lima IP, Moratelli R, Tavares V, da C, Gregorin R et al Checklist of Brazilian bats, with comments on original records. Check List. 2014 10.15560/10.4.808

[6] Lima FEDS, Campos FS, Kunert Filho HC, Batista HBDCR, Carnielli Júnior P, Cibulski SP et al Detection of Alphacoronavirus in velvety free-tailed bats (Molossus molossus) and Brazilian free-tailed bats (Tadarida brasiliensis) from urban area of Southern Brazil. Virus Genes. 2013 10.1007/s11262-013-0899-x 23504146 PMC7089283

[7] Asano KM, Hora AS, Scheffer KC, Fahl WO, Iamamoto K, Mori E et al Alphacoronavirus in urban Molossidae and Phyllostomidae bats. Brazil Virol J. 2016 10.1186/s12985-016-0569-4 27342195 PMC4920988

[8] Góes LGB, Ruvalcaba SG, Campos AA, Queiroz LH, de Carvalho C, Jerez JA et al Novel bat coronaviruses. Brazil and Mexico Emerging Infectious Diseases. 2013 10.3201/eid1910.130525 24050144 PMC3810755

[9] Corman VM, Rasche A, Diallo TD, Cottontail VM, Stöcker A, de CD SBF et al Highly diversified coronaviruses in neotropical bats. J Gen Virol. 2013 10.1099/vir.0.054841–0 23761408

[10] Simas PVM, DE Souza Barnabé AC, Durães-Carvalho R, De Lima Neto DF, Caserta LC, Artacho L et al Bat coronavirus in Brazil related to appalachian ridge and porcine epidemic diarrhea viruses. Emerg Infect Dis. 2015 10.3201/eid2104.141783 25811911 PMC4378475

[11] Góes LGB, Campos AC de A, Carvalho C de, Ambar G, Queiroz LH, Cruz-Neto AP, et al Genetic diversity of bats coronaviruses in the Atlantic Forest hotspot biome, Brazil. Infect Genet Evol. 2016 10.1016/j.meegid.2016.07.034 27473780 PMC7106056

[12] Lee K, Brumme ZL. Operationalizing the One Health approach: The global governance challenges. Health Policy Plan. 2013 10.1093/heapol/czs127 23221123

[13] Halliday JEB, Allan KJ, Ekwem D, Cleaveland S, Kazwala RR, Crump JA. One health: Endemic zoonoses in the tropics: A public health problem hiding in plain sight. Vet Rec. 2015 10.1136/vr.h798 25722334 PMC4350138

[14] Destoumieux-Garzón D, Mavingui P, Boetsch G, Boissier J, Darriet F, Duboz P et al The one health concept: 10 years old and a long road ahead. Frontiers in Veterinary Science. 2018 10.3389/fvets.2018.00014 29484301 PMC5816263

[15] Tilocca B, Soggiu A, Musella V, Britti D, Sanguinetti M, Urbani A et al Molecular basis of COVID-19 relationships in different species: a one health perspective. Microbes Infect. 2020 10.1016/j.micinf.2020.03.002 32194253 PMC7102648

[16] Ellwanger JH, Kaminski V de L, Chies JAB. How to detect new viral outbreaks or epidemics? We need to survey the circulation of viruses in humans and other animals using fast, sensible, cheap, and broad-spectrum methodologies. Braz J Infect Dis. 2017 10.1016/j.bjid.2016.12.001 28012932 PMC9427588

